# Acute Myocarditis Presenting as Acute Coronary Syndrome

**DOI:** 10.7759/cureus.5212

**Published:** 2019-07-23

**Authors:** Christian M Mosebach, Varun Tandon, Manish Kumar

**Affiliations:** 1 Internal Medicine, University of Connecticut Health Center, Farmington, USA; 2 Internal Medicine, University of Arizona College of Medicine - Phoenix, Phoenix, USA

**Keywords:** complete heart block, ventricular arrhythmias, acute coronary syndrome, acute myocarditis

## Abstract

A 50-year-old male presented to the hospital with an approximate three-week history of nausea, fever, and back pain. Upon initial evaluation he had an electrocardiogram with ischemic changes and initial labs significant for a troponin of >25.0 ng/ml (<0.30 ng/ml), pro b-type natriuretic peptide (proBNP) of 9884 pg/ml (<125 pg/ml), and a lactic acid of 4.3 mmol/L (0.5-1.9 mmol/L). There was a concern for an acute coronary syndrome presenting as cardiogenic shock, but the patient was unable to tolerate left heart catheterization. He had a rapid clinical decline and despite all efforts, he passed away. The initial cause of death was thought to be due to an acute myocardial infarction, however, autopsy results were consistent with acute myocarditis. This case highlights the presentation of acute myocarditis as an acute coronary syndrome with complete heart block.

## Introduction

Inflammation of the heart was first investigated by Jean Baptiste Senac, a physician in Versailles, France in 1749. However, the medical term "myocarditis" was not found in literature until 1837. Initially, the term "myocarditis" referred to multiple different types of cardiomyopathies [1]. It was not until the 1980’s that the World Health Organization, along with the International Society and Federation of Cardiology, attempted to differentiate between myocarditis and all other etiologies that could cause cardiomyopathy [2].

Myocarditis is defined as an inflammatory disease of the cardiac myocytes [1]. The disease can be broadly classified as fulminant, acute, chronic active, or chronic persistent. There is a distinct onset of a viral prodrome within the prior two weeks before heart failure symptoms develop in patients with fulminant myocarditis. This distinct onset differentiates fulminant myocarditis from acute myocarditis, the latter having a more prolonged onset of symptoms. Although the onset of symptoms in fulminant myocarditis is more abrupt, patients with fulminant disease generally have a better long term prognosis as compared to patients with acute myocarditis [3]. Myocarditis is a relatively rare disease with studies showing a global annual prevalence of approximately 22 people for every 100,000 [4].

## Case presentation

**Figure 1:**
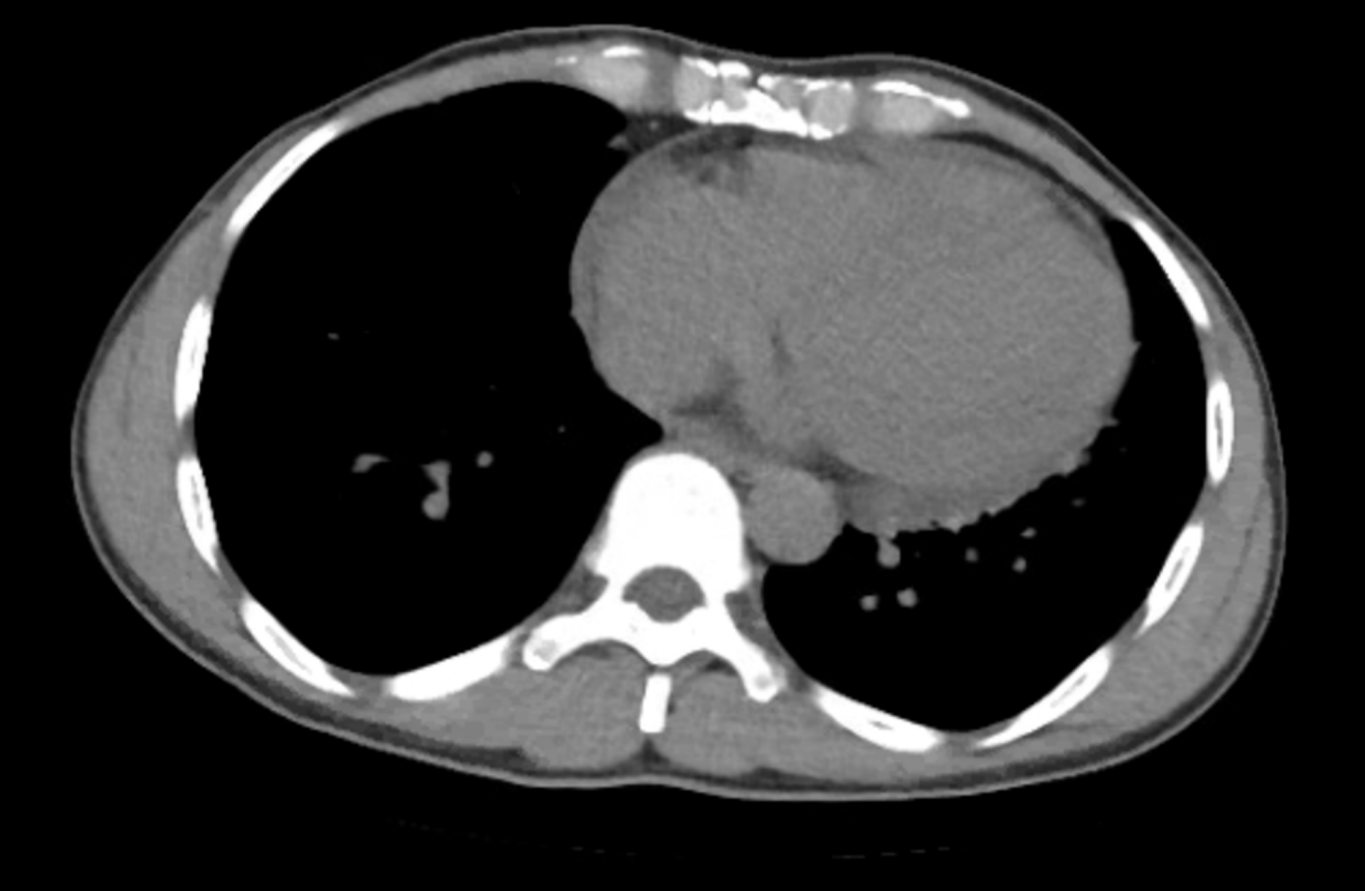
Admission computed tomography (CT) scan showing cardiomegaly

**Figure 2:**
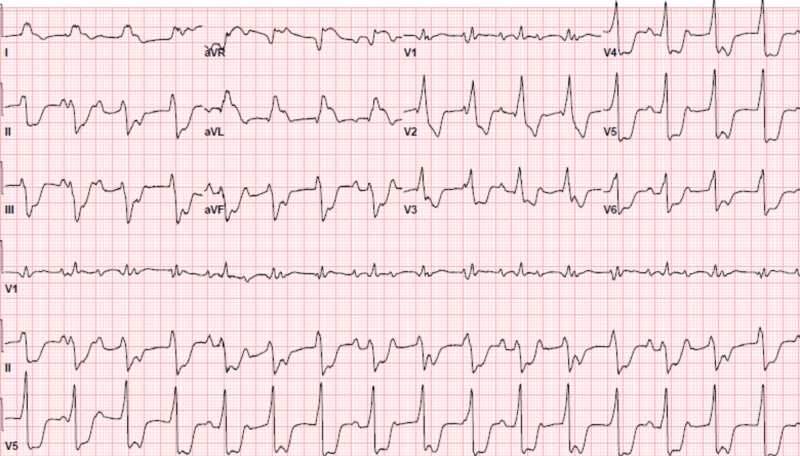
Admission electrocardiogram

A 50-year-old male with a past medical history of chronic musculoskeletal back pain and depression presented to the emergency department with a three-week history of fever, nausea, and worsening back pain. He was initially diagnosed with a musculoskeletal back strain and sent home from the emergency department. The patient returned to the hospital a few days later with similar symptoms. On exam, he was in distress, had elevated jugular venous pressure, bibasilar crackles on lung auscultation, and his extremities were cool and his skin was diaphoretic. A computerized tomography (CT) of the abdomen/pelvis without contrast was performed to evaluate for possible renal calculi. There were no identified calculi, however, he did have a new finding of cardiomegaly with small pericardial effusion (Figure 1). An electrocardiogram (ECG) showed a complete heart block with a junctional escape rhythm, ST segment elevation in leads I, aVL, aVR, and diffuse ST segment depression in the other leads (Figure 2). A bedside transthoracic echocardiogram revealed global hypokinesis with an estimated ejection fraction of 20%. Initial laboratory results were significant for a troponin of >25.0 ng/ml (<0.30 ng/ml), pro b-type natriuretic peptide (proBNP) of 9884 pg/ml (<125 pg/ml), and a lactic acid of 4.3 mmol/L (0.5-1.9 mmol/L). His physical exam along with these findings was thought to be most consistent with an acute myocardial infarction presenting with cardiogenic shock. Unfortunately, the patient was not able to tolerate left heart catheterization because he was unable to remain still and repeatedly contaminated the sterile field. 

**Figure 3:**
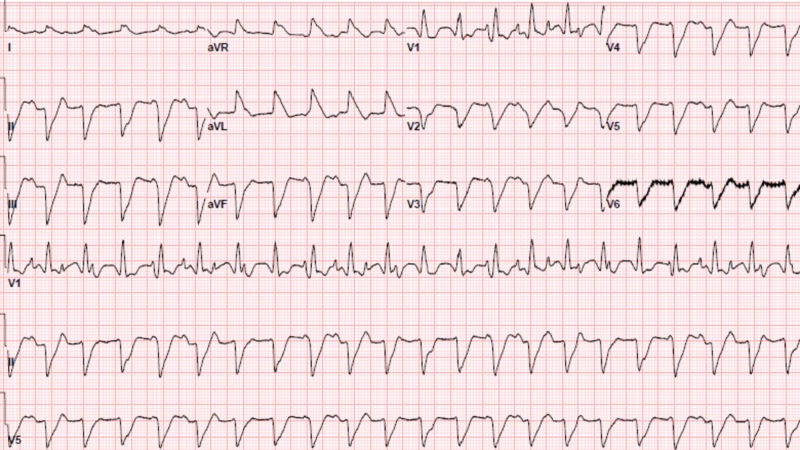
Electrocardiogram showing monomorphic ventricular tachycardia

Due to his rapid clinical decline within the first day of hospital admission, he was admitted to the cardiac intensive care unit. He subsequently went into a monomorphic ventricular tachycardia (VT) (Figure 3). The patient was given intravenous Lidocaine 100mg and was started on a Lidocaine drip, had a transvenous pacer placed, and had synchronized cardioversion performed at 150 and 200 joules. Despite all of these interventions, he remained in monomorphic VT. The patient decompensated and went into pulseless VT and advanced cardiac life support (ACLS) was initiated. Despite 45 minutes of ACLS he remained in pulseless VT and after discussion with the family resuscitation efforts were discontinued. The cause of death was attributed to an acute myocardial infarction. Interestingly, autopsy results determined that his cause of death was due to acute myocarditis.

## Discussion

When the patient went into monomorphic VT he had a transvenous pacer placed. This was done because he initially presented with a third-degree heart block causing concern that after synchronized cardioversion he could have symptomatic bradycardia if he did not go into a junctional escape rhythm. If he developed symptomatic bradycardia he would have required transvenous pacing to maintain adequate hemodynamics. Due to the patient's rapid clinical decline and inability to tolerate a left heart catheterization, an endomyocardial biopsy was not performed. 

At the time of autopsy, microscopic examination of his myocardium showed inflammation throughout both ventricles and atria. The coronary arteries were also examined with the only significant finding being 40% stenosis of the left anterior descending coronary artery. The patient’s non-specific back pain, nausea, and fever in the approximate three weeks prior to hospital presentation was likely a viral infection that precipitated his episode of acute myocarditis. Unfortunately, no viral-specific treatment modality has shown to improve survival free from heart failure. The treatment for patients with acute viral myocarditis includes the standard pharmacological and mechanical treatments used to treat other etiologies of heart failure [5].

The gold standard test to perform on patients with suspected myocarditis is an endomyocardial biopsy. There are two clinical scenarios in which an endomyocardial biopsy has class I, level B evidence (strong recommendation, moderate-quality evidence). The first scenario is new-onset heart failure within the last two weeks with a normal-sized or dilated left ventricle and associated hemodynamic compromise. The second scenario is new-onset heart failure over the last two weeks to three months with a dilated left ventricle and new ventricular arrhythmias, high degree heart block, or failure of the patient to respond to standard therapy within one to two weeks [6]. 

Despite their critically ill presentation, patients with fulminant myocarditis often have a good prognosis if they can survive their initial hospital presentation. One study identified 147 patients (132 acute myocarditis and 15 fulminant myocarditis) looked at long-term outcomes in patients and found that 93% of patients with fulminant myocarditis were alive and did not require a heart transplant 11 years after initial diagnosis as compared to only 45% of patients with acute myocarditis [7].

## Conclusions

Myocarditis is a rare disease that is most commonly due to a viral etiology. Its presentation is variable, however, myocarditis can sometimes present with elevated troponins and ischemic changes on the electrocardiogram. Although it is a rare condition, it is important to have myocarditis on the differential for any patient who presents with the acute coronary syndrome as both conditions can mimic one another.

## References

[1] Fung G, Luo H, Qiu Y, Yang D, McManus B (2016). Myocarditis. Circ Res.

[2] Richardson P, McKenna W, Bristow M (1996). Report of the 1995 World Health Organization/International Society and Federation of Cardiology Task Force on the definition and classification of cardiomyopathies. Circulation.

[3] Cooper LT (2009). Myocarditis. N Engl J Med.

[4] Vos T, Barber RM, Bell B (2015). Global, regional, and national incidence, prevalence, and years lived with disability for 301 acute and chronic diseases and injuries in 188 countries, 1990-2013: A systematic analysis for the global burden of disease study 2013. Lancet.

[5] Kindermann I, Barth C, Mahfoud F (2012). Update on Myocarditis. J Am Coll Cardiol.

[6] Cooper LT, Baughman KL, Feldman AM (2007). The role of endomyocardial biopsy in management of cardiovascular disease. Circulation.

[7] McCarthy RE, Boehmer JP, Hruban RH (2000). Long-term outcome of fulminant myocarditis as compared with acute (nonfulminant) myocarditis. N Engl J Med.

